# Exploring Smart Glasses for Augmented Reality: A Valuable and Integrative Tool in Precision Livestock Farming

**DOI:** 10.3390/ani9110903

**Published:** 2019-11-01

**Authors:** Maria Caria, Gabriele Sara, Giuseppe Todde, Marco Polese, Antonio Pazzona

**Affiliations:** Department of Agricultural Science, University of Sassari, 07100 Sassari, Italy; mariac@uniss.it (M.C.); 30029770@studenti.uniss.it (G.S.); 30048795@studenti.uniss.it (M.P.); pazzona@uniss.it (A.P.)

**Keywords:** remote assistance, QR code scanning, dairy sheep, mobile augmented reality, precision farming, head wearable device, animal feeding, animal breeding, remote learning

## Abstract

**Simple Summary:**

Introducing new technologies in the agricultural and livestock field does not always lead to straightforward on-farm activities. Smart glasses for augmented reality are a new technology that may assist workers in many operations, allowing them to visualize, in the glasses’ lens, diverse information related to a single subject (e.g., animal, plant, feed stock, machinery) or to receive assistance in real-time through video-calls. Using commercially available smart glasses, we explored their potential usefulness in livestock farms. The device was tested using all the functions available in different conditions, both in laboratory and open field environments. The results obtained highlighted the important contribution to assist workers in on-farm daily activities, thanks to the clear and rapid data visualization and to the good quality of audio-video streaming. Specifically, smart glasses enable real time file consulting, data collection, data sharing and remote assistance, all done while working hands-free.

**Abstract:**

The growing interest in Augmented Reality (AR) systems is becoming increasingly evident in all production sectors. However, to the authors’ knowledge, a literature gap has been found with regard to the application of smart glasses for AR in the agriculture and livestock sector. In fact, this technology allows farmers to manage animal husbandry in line with precision agriculture principles. The aim of this study was to evaluate the performances of an AR head-wearable device as a valuable and integrative tool in precision livestock farming. In this study, the GlassUp F4 Smart Glasses (F4SG) for AR were explored. Laboratory and farm tests were performed to evaluate the implementation of this new technology in livestock farms. The results highlighted several advantages of F4SG applications in farm activities. The clear and fast readability of the information related to a single issue, combined with the large number of readings that SG performed, allowed F4SG adoption even in large farms. In addition, the 7 h of battery life and the good quality of audio-video features highlighted their valuable attitude in remote assistance, supporting farmers on the field. Nevertheless, other studies are required to provide more findings for future development of software applications specifically designed for agricultural purposes.

## 1. Introduction

At the beginning of the 1990s, Milgram and Kishino [1] introduced the model of the virtuality continuum, characterized by the real environment and the virtual environment on the outer limits of the continuum. Among these limits, there is the mixed reality where real and virtual objects coexist at different levels on the continuum. Mixed reality includes augmented reality (AR) and augmented virtuality. The former is close to the real environment and the latter to the virtual world. Therefore, AR enriches the real world vision with virtual objects whereas augmented virtuality enlarges the virtual world vision with real objects [1].

Later, the term augmented reality was associated with all those technologies that are characterized by the following three features: combine physical and virtual objects over the real environment; interact in real time; align physical and virtual objects with each other [2]. Therefore, an AR system allows the overlaying of different virtual elements, generated by a computer (text, chart, audio, video, image, etc.) over the real world giving information about the physical elements that our senses could not provide [3].

The AR systems are associated and implemented in different devices, such as personal computers, head mounted devices, smartphones, tablets and so forth. In fact, AR is a combination of several hardware and software technologies that work together to bring digital information in the visual field. 

The recent innovation in mobile technology and wireless networking, allowed the development and improvement of mobile AR. It represents a useful system which allows users to receive and interact with augmented information everywhere, which otherwise would not be available without a stationary position [4]. 

Nowadays, mobile AR is mainly based on smartphones (hand-held devices) but might be replaced by smart glasses (SG), which are hands-free systems and might have great potential to become the main platform for AR [5].

SG are head-worn miniature computers, provided by a display in front of the user’s eyes, representing the main discriminating feature. The augmented overlay contents can be shown on the display with three different optics systems—video, optical and retinal see-through. The first one combines the real and virtual view in one completely digital user’s vision, the second one overlays the virtual objects straight to a real world user’s vision and the last one, with low-power laser light, projects the virtual objects directly onto the retina. Smart glasses are also commonly provided with a camera for image acquisition, sensors (GPS, accelerometer, gyroscope, etc.) and input controls (gesture, voice) [6].

AR systems are applied in many different areas mainly in education, manufacturing, medicine, tourism and entertainment [7,8,9,10,11,12,13]. There is an increasing interest in this matter attested by the continuously growing number of research articles and conference papers published on AR so far. As shown by the bibliographic data analysis in the Scopus database, the published documents increased from one hundred in 1997 to over three thousand in 2018.

In the agricultural sector, one of the first research works on AR applications was published by King et al. [14] concerning the development of an AR system to provide information on the grape field. Other authors investigated the importance of the AR technology in agriculture, focusing on fertilizing and spraying operations [15], weed identification [16], greenhouse management [17] and farmer management support [18,19]. Additionally, Cupial [20] underlined the importance and the great potential of AR in the agricultural field. However, only a few of these studies are focused on wearable AR systems and its potentiality to support and improve farmer activities [15,21,22]. Furthermore, no research is still carried out on the application of smart glasses for AR in the livestock sector, especially in the growing field of the technologies used in Precision Livestock Farming (PLF).

Smart glasses might represent an important instrument, complementary to other tools used in PLF, to make available substantial information to farmers in real time. In fact, augmented reality will soon become popular, especially since there are many areas in which its implementation will be advisable [20]. Recent studies have underlined how the implementation of precision technologies in livestock farms improved animal welfare and management [23,24,25,26], farm profits and environmental sustainability [27,28,29]. Likewise, Todde et al. [30] showed a positive association among technological investments and a farm’s energy efficiency.

Smart glasses for AR are closely related to PLF principles [31] since they could help farmers to manage the animal husbandry precisely. These technologies might easily provide useful information on the individual head about its identification, health status, productivity, feed ration and so forth.

Another important aspect regarding the use of SG in agriculture is related to farmer operations, which requires the use of both hands. Therefore, using a hands-free system information on plants, animals and soil can be obtained while working [22].

The aim of this study was to evaluate the performances of an augmented reality head-wearable device as a valuable and integrative tool in precision livestock farming.

The research questions to be answered by this study were: (i) which are the possible applications of the smart glasses in livestock farming; (ii) which could be the smart glasses utility in the farm management considering the available functions; (iii) how do the smart glasses perform during on-farm operations.

## 2. Materials and Methods

**Figure 1:**
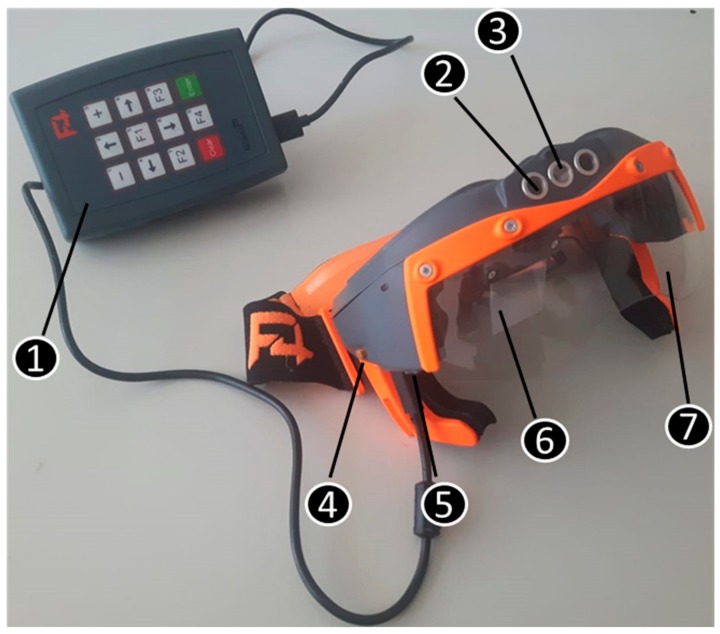
GlussUp F4 smart glasses adopted in this study: (1) joypad; (2) video/photo camera; (3) front light; (4) smart glasses right side button; (5) audio jack 3.5; (6) combiner to visualize the augmented contents; (7) frontal protection lens.

In this study, the GlassUp F4 Smart Glasses (F4SG), produced by an Italian company (GlassUp, Modena, Italy) were adopted. F4SG are AR viewers (Figure 1), mainly designed for industrial use, that work in pairs with a remote control Dashboard (management software to be installed in computers).

The F4SGs are coupled with an external joypad, which allows accommodation of the battery pack and control of the glasses with navigation buttons (enter, clear and arrows up, down, right, left) and five function keys that can be set with different tasks (front light, photo capture, video recording and scan-code). In one side of the joypad there are a series of led indicating the battery state of charge (4 blue and one red), the Wi-Fi connection and the Bluetooth status (connected or not connected). Furthermore, on the right side of the glasses, there is another button that can be set with one of the commands listed above. The external joypad contains the Wi-Fi system that was used to connect the smart glasses to the phone and to internet. In Table 1, the main features of the tested smart glasses are reported.

During the study, some F4SG functions, such as QR (Quick Response) code scanning, VoIP (voice over internet protocol) call and video streaming, were tested in the laboratory and in a real work environment to investigate the possible applications of the smart glasses in the agricultural field and specifically in livestock farms.

### 2.1. Laboratory Tests

Several tests were performed in the laboratory of the Department of Agricultural Science at the University of Sassari, to assess the implementation of smart glasses for AR in livestock farming. The performance of QR code scanning function was evaluated. The trials were carried out simulating the main activities accomplished in livestock farms (milking, feeding, breeding, etc.). The on farm information used in these tests were incorporated on QR codes. The QR code, generally organized as black squares arranged in a square grid on a white background, holds a wide storage capacity enabling the encoding of many different types of information [32]. This type of code has a high correction rate, meaning that the QR code can be read correctly even if a quota of the symbol is tainted or torn [32,33]. These features make the QR code suitable for application in agricultural contexts.

#### 2.1.1. QR Code Scanning Time

The QR code scanning time and battery life were tested in continuous and in repeated measurements. The QR code scanning time, considered as the time frame from activation of the scan-code function to the visualization of the associated file, was monitored. The scan-code function was associated with the right side button of smart glasses. The trials consist of a continuous QR code scanning and file opening until the battery was completely discharged. For this test, 24 QR codes of three different sizes were printed with the following dimensions—3.5 cm (series 1), 4 cm (series 2), 7.5 cm (series 3) and with 25 modules per side. The series was placed on a vertical plane at 33 cm of distance between each single QR code and at a height of 160 cm, simulating position and height of the animals during milking operation. The QR codes scans were carried out by three different operators (named a, b and c) at a scanning distance of about 40 cm. The test was repeated for three times and the battery was completely recharged before the beginning of each test. The total number of QR codes scanned were 3489.

**Figure 2:**
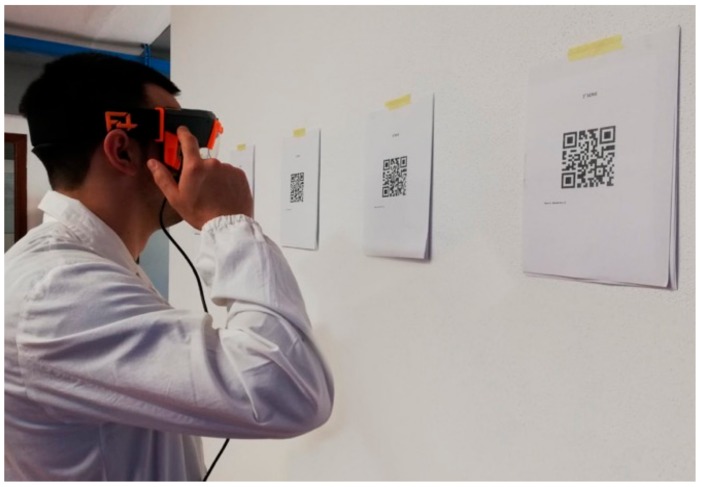
Laboratory tests: Quick response (QR) codes scanning procedure with a 7.5 cm code size.

The scanning time was monitored with a chronometer, which was started as soon as the operator activated the scan-code function on F4SG and stopped when the file opened up (Figure 2).

#### 2.1.2. QR Code Scanning Distance

An evaluation on the QR code size in relation to optimal and maximum scanning distance was carried out. The optimal scanning distance refers to the distance at which the operator is able to scan and open the file immediately, while the maximum one is the distance beyond which the QR code is not detected by the smart glasses camera. Six QR codes of different dimensions were used—1.5 × 1.5 cm, 3.5 × 3.5 cm, 4 × 4 cm, 7.5 × 7.5 cm, 13 × 13 cm and 20 × 20 cm all of these with 25 modules per side. The measured distance was between the vertical plane, on which the QR code is located (160 cm height) and F4SG camera. Furthermore, a linear regression between QR size and scanning distance was assessed.

#### 2.1.3. QR Code and Farm Information Sheet

A Microsoft Word file was adopted to develop the Farm Information Sheet (FIS), which contained the information about the animals (breed, animal ID, group, lactation days etc.) and feed stocks (feed type, amount, quality, harvesting time, etc.) available at the farm level. A large amount of information can be reported for these issues, making the FIS hard to retrieve at a glance. For this reason, based on farmers’ suggestions, we selected the most important information that should be available to the farmers on the SG. These records were outlined on a single page to allow the user to visualize immediately all the information without using the joypad. The FIS was written in Time New Roman with a font size of 18 points. Moreover, a milk emission flow curve was included in the FIS and uploaded, as a pdf file, in the F4SG memory. Each FIS was associated with a unique QR code.

Using the scan-code function of the F4SG (activated using the key on the right side of the glasses) the user is able to scan, open and visualize the FIS on the smart glasses display.

### 2.2. Audio-Video Quality Tests

#### 2.2.1. Video Call Lag Time

**Figure 3:**
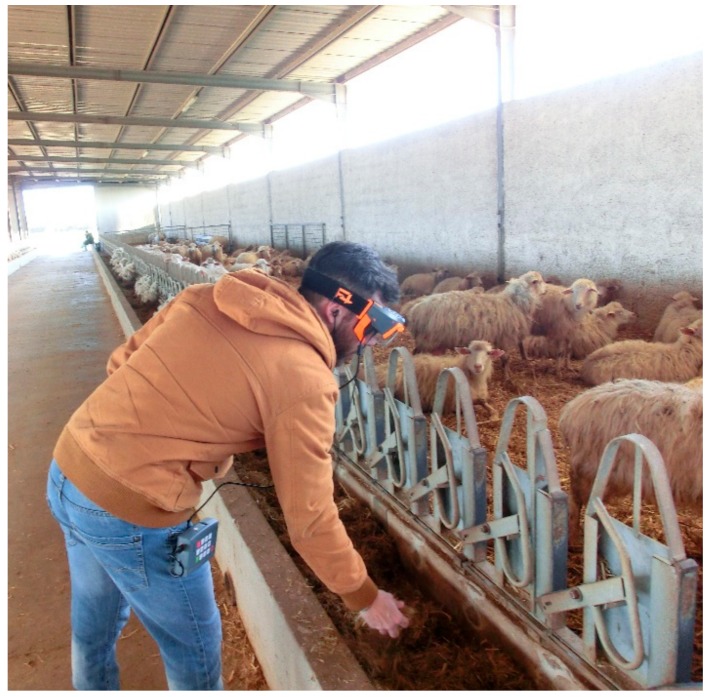
While working usage of the smart glasses hands-free device in the paddock.

The lag time of a remote video call between F4SG and laptop (dashboard) was evaluated (Figure 3). The test was carried out with the emitting operator (EO) wearing the smart glasses (placed in the livestock farm) and the receiving operator (RO) that was the person using the laptop (placed in the lab of the Department of Sassari). Smart glasses and laptop were connected using 4G mobile phone (Huawei P20 lite) network internet tethering. The audio-video lag time between the EO and RO was evaluated by synchronizing the operators clocks and recording the emission and receiving time of a predetermined signal for audio and a predetermined position for video [34]. The lag time test was carried out with two different streaming qualities, 4G and 3G and for each quality and types of signal (audio or video) were recorded 16 times both for EO and RO.

#### 2.2.2. Vision Testing Through F4 Smart Glasses Via Remote Internet Connection

A vision quality test was carried out to evaluate the quality of the transmitted image through the F4SG. A standard Snellen vision test charts were performed. The test was achieved with two different streaming qualities (3G and 4G). The EO was placed at 50 cm distance from the Snellen chart [34] and the RO read the characters in the 16-inch laptop monitor.

### 2.3. Battery Life

During the QR code scanning and video call, tests also monitored the battery life. In the first case, checking its status at the end of each scanning series (24 QR codes), and in the second one checking the status during a continuous VoIP call, monitoring the battery LED on the side of the joypad.

### 2.4. Farm Tests

**Figure 4:**
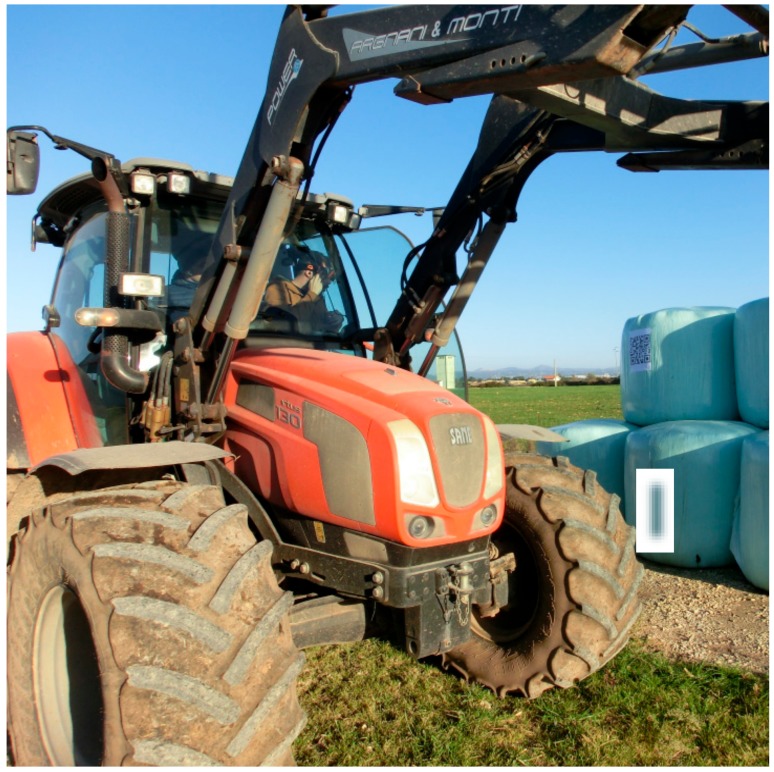
On-farm QR code scanning procedure tests. The operator with smart glasses was placed at different distances, heights and positions in the tractor cabin while selecting the appropriate bale silage for feed preparation.

The F4SG were utilized in a dairy sheep farm in Sassari (Sardinia, Italy). The audio-video quality of the transmitted contents from the smart glasses to the Dashboard was evaluated. In fact, the background noise and different level of light exposure, in real work environments, may affect the audio-video comprehension and the QR code readability. The tests were carried out with the RO that was located in the lab of the Department and the EO which was transmitting from the sheep shed and milking parlour. For this test 3G streaming quality was used. Furthermore, the scan-code function was tested with the QR code positioned, respectively, on bale silage and on the sheep’s tail. This enabled to provide information in real time on the features and composition of the feed (type of forage, humidity, harvesting data, etc.) and for the individual animal. Two 28 × 28 cm QR codes were attached to the bales silage, placed at 200 and 90 cm height and scanned from 3.0 to 5.0 m distance from the ground and from the tractor cabin (Figure 4).

On the sheep’s tail were attached 3.5, 4 and 7.5 cm QR codes, placed at 160 cm height and scanned from 50 cm distance.

### 2.5. Statistical Analysis

Descriptive statistics (arithmetic average, standard deviation, minimum and maximum) were calculated for the scanning time in relation to the QR code size and operators. The variations in the parameters were also analysed by evaluating frequency distributions. Statistical analysis was carried out by comparing the scanning time within different QR codes size (3.5 cm, 4 cm, 7.5 cm) and within three operators (a, b, c), using the Mann-Whitney U test from the R Studio software (version: 3.4.4).

## 3. Results

The study showed results for commercial smart-glasses GlassUp F4, which were stressed to determine their performance in extreme conditions and evaluated for possible applications in livestock farming operations.

### 3.1. QR Code Scanning Time

The purpose of this trial was to estimate the opening times linked to the use of the QR scanning function. The importance of this test is underlined by the fact that preliminary evaluations can help understanding of which agricultural and livestock activities this new technology can be integrated with.

The results obtained during the scanning of three different QR code dimensions are shown in Table 2. In all cases there is a wide range between minimum and maximum scanning time (30.6, 29.7 and 24.4). The size of the QR codes significantly influenced the scanning time (*p* < 0.001) regardless of the distance of the operator. Specifically, the F4SG had minor response time when scanning QR codes with 7.5 cm size (7.7 s), rather than other small dimensions, 8.6 and 11.0 s respectively for 4.0 and 3.5 cm. The difference between the smaller and the bigger codes was, on average, 3.3 s. This time was negatively perceived by the participants, even if in real work conditions 3 s may not affect the working time.

The results related to the operators’ performance are summarized in Table 3. QR code scanning time was significantly different between operators “a” and “b” and between “b” and “c.” There are no differences between operators with higher average values. All operators had, on average, comparable minimum and maximum values of scanning time (about 4.1 s and 33.8 respectively).

**Figure 5:**
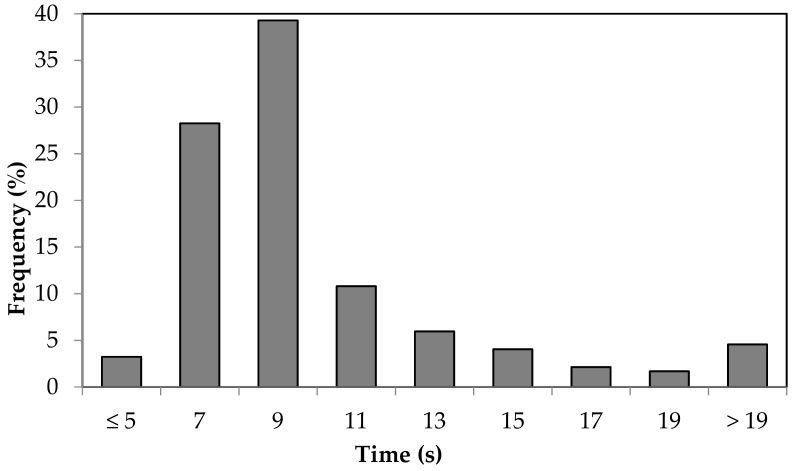
Frequency distribution diagram of QR codes scanning times.

There is a wide distribution in QR code scanning time frequency. In 67% of cases, the scanning time was between 5.1 s and 9 s (Figure 5). Only 3 % of the scanned codes occurred after 5 s or less, while 5% took more than 19 s. The maximum time recorded during the tests corresponded to 34.7 s and although smart-glasses enabled hands-free working, excessive response time of scanning procedures significantly increase the operating time.

### 3.2. QR Code Scanning Distance

Using QR codes in livestock farming may meet two main limitations related to their size and the scanning distance. In fact, the surface available for supporting QR code may be limited (i.e., animal body) and similarly, the reading distance of QR codes could be strictly linked to the farm activities (i.e., selecting the feed from the tractor cabin).

**Figure 6:**
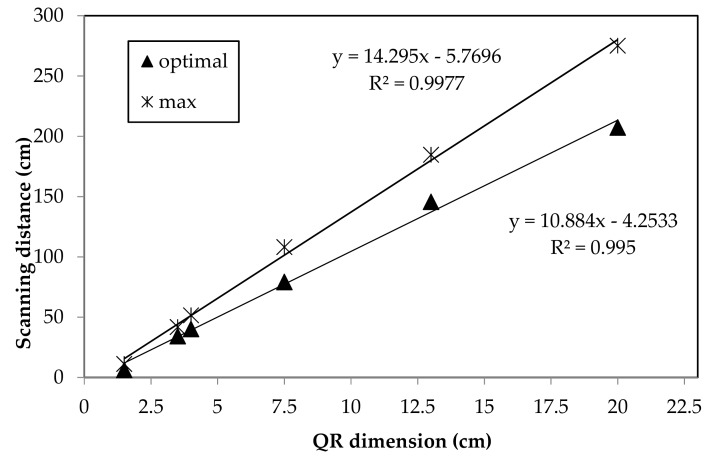
Correlation between QR code size and scanning distance, optimal (▲) and maximum (×).

The correlations among measured scanning distance and QR code dimensions are shown in Figure 6. The results highlighted that the optimal and the maximum scanning distances are linearly correlated with the code size. There was a high relationship between maximum (R^2^ = 0.997) and optimal (R^2^ = 0.995) scanning distance with the QR code size. The optimal distance resulted in ten times the QR code size and thirteen times the maximum one. Likewise, other authors [35] confirmed that the QR code readability was affected by the interaction of scanning distance and code size. Bigger QR code size allows more scanning points than the smaller one. Moreover, the readability of QR codes with standard print resolution features (4 × 4 pixels per module with 300 dots per inch) is possible with common optical devices [35].

**Figure 7:**
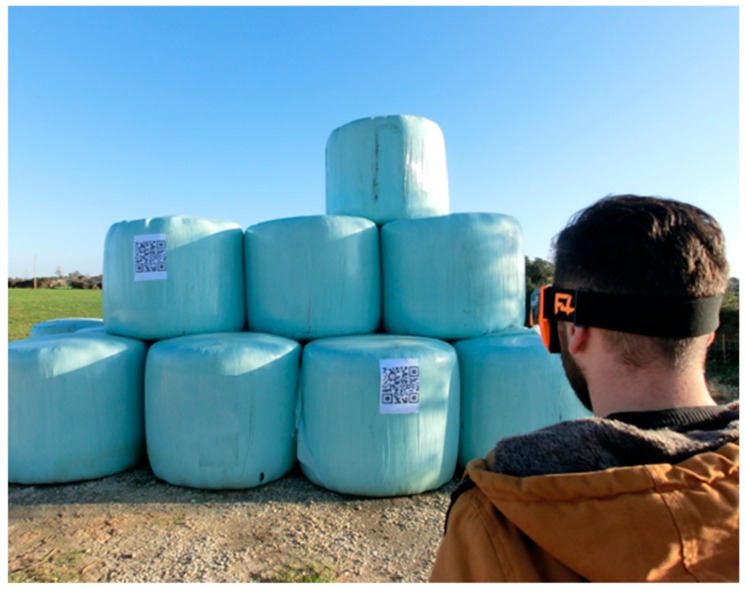
Farm scanning test to detect feed information sheet on the bale silage. The QR code of 28 × 28 cm size placed at 90 and 200 cm height.

The results obtained during the feeding procedures at the farm, while working from the ground or with the tractor, showed that a distance higher than about 4.0 m did not allow retrieval of the information on smart glasses (Figure 7). Nevertheless, 3.0 m distance from the QR code to the device enabled a prompt response.

### 3.3. Battery Life

Battery drain while working can be a serious issue to deal with. Likewise, knowing the amount of information achievable in terms of the amount of scanning by means of smart glasses will optimize the work process. In order to qualify the battery life of the F4 glasses, two tests were performed considering different operative applications. The results of the battery life are listed in Table 4.

### 3.4. Audio-Video Quality

The audio-video lag time was calculated with two different streaming qualities (3G and 4G). In both cases, a lag time was registered between the emitting and receiving signal. The video lag time was 2.13 and 2.28 s, respectively for 3G and 4G. Instead, the audio lag time was lower (0.67 s) for the 4G than the 3G streaming quality (1.81 s).

**Figure 8:**
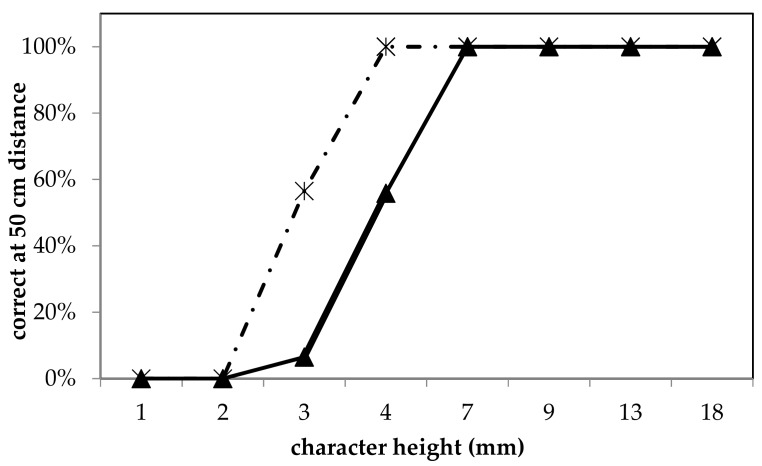
Snellen chart vision test, describing the percentage of correct letter individuation in relation to its dimension. The two streaming video qualities are reported 3G (continuous line) and 4G (dashed line). The test was performed through F4 smart glasses at 50 cm distance from the chart. Reading was carried out on a 16 inches display.

Setting the 4G streaming quality in F4SG allowed the receiving operator to improve the reading performance of the Snellen chart video-transmitted (Figure 8). In fact, with the 4G streaming quality, all characters 4 mm height or larger were correctly identified, while reading the line 3 mm an average of 55.5% of letters were recognized. The 3G streaming quality allowed us to correctly read (100% of the cases) the 7 mm characters’ height or larger, while 6 and 5 mm height were legible in the 55.9 and 6.5% of the trials, respectively. The character height 2 mm or smaller was illegible in all of the trials.

**Figure 9:**
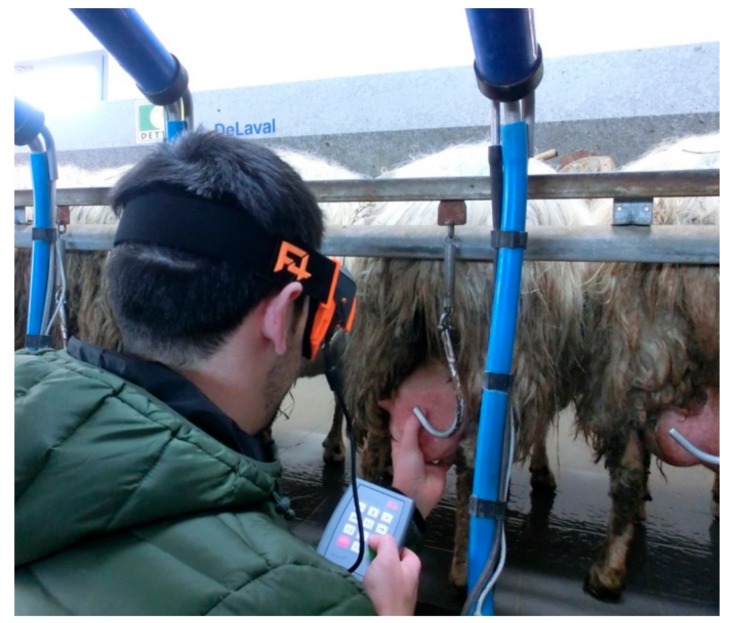
Testing remote assistance smart glasses’ performances during mammary gland inspection.

The outcomes related to the video performance carried out during the farm tests underlined the high quality necessary to document all the on-farm related circumstances. Difficult lighting conditions may compromise the video quality. However, during the trials conducted on milking parlour, that is, in a low lighting environment, did not affect the device and operator performances (Figure 9).

The audio quality of F4SG was performed at different intensity levels of background noise at the farm. One of the most critical circumstances was found while driving a tractor, followed by working in the sheep shed and during milking. However, the conversations between the emitting and receiving operators were fluent and without misunderstandings. Specifically, emitting and receiving operators did not have any difficulties hearing every sentence while speaking at normal volume.

## 4. Discussion

The study provided results of the use of commercial smart-glasses GlassUp F4, which were stressed to determine their performances in extreme conditions and evaluated for possible applications in livestock farming operations. In agricultural and livestock tasks, specific information per subject is commonly needed (e.g., animal, plant, orchard row, field, greenhouse, etc.). Using F4SG to retrieve information in several on-farm activities, which were previously loaded (as a file) onto the memory of the device. One or more pages of information can be visualized, scrolling through different pages using manual control, as there is no verbal scrolling command pre-programmed into the current version of SG. However, the use of manual commands instead of vocal commands seems to be the optimal solution, due to the presence of background noises in many different farming contexts [36].

**Figure 10:**
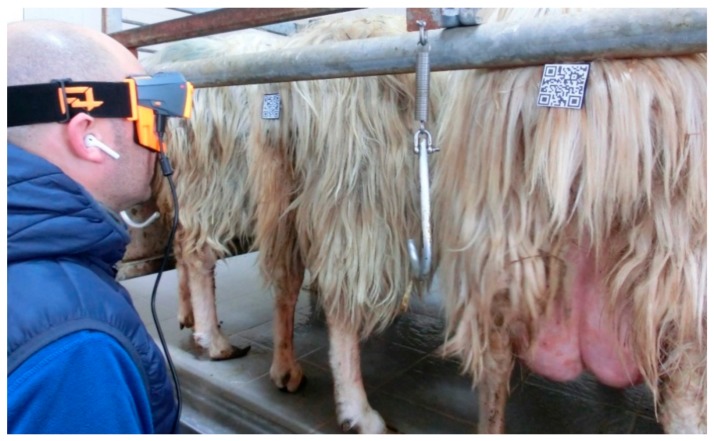
Scanning QR codes positioned on the sheep’s tail in self-locking yokes at the milking parlour.

The subjects must have an identification code (i.e., QR code) to be detected by the operator. The trials on the scan of QR codes with different dimensions at the same position were effectuated to underline the importance of it on the scan operative time. It is important to accurately choose the size of QR code, especially when the scanning occurs on animals standing in self-locking yokes, in milking parlours or in cattle sheds (Figure 10). In fact, as found in this study, scanning time with SG was inversely proportional to QR code size. The distance of the QR code is fundamental to optimize the scanning process in terms of time and operator comfort [35,37].

The influence of the operator while using smart glasses was clear; significant differences among the three average scanning times were observed in this study. Several works explained the different aptitude and acceptance of users for modern technologies [38,39,40,41,42], which probably affected the scanning times of this study.

The response time found among the three QR code series did not appear considerable because the operator could continue to work since the information is visualized hands-free on a head-worn display. For this reason, the response time should not be considered dead-time. In fact, scanning time may seem long but we have to consider that into this time frame several steps are coupled, specifically, scan-code function activation, QR code scanning, file opening and file visualization. The only step that needs the attention of the operator is related to the QR code scanning. During the other steps, the operator is able to accomplish the tasks without staring at the combiner (SG display). Moreover, watching the optical display of smart glasses is not comparable to looking at a monitor. In fact, the information is projected into a lens positioned few centimetres from the user’s eye. This allows the user to see through the document displayed, thanks to the human ability to focus on objects at different distances.

In recent years, technology progress has accustomed users to faster connections and rhythms. As a consequence, the response time observed in this study may be negatively perceived by the operator when using SG for the first time.

**Figure 11:**
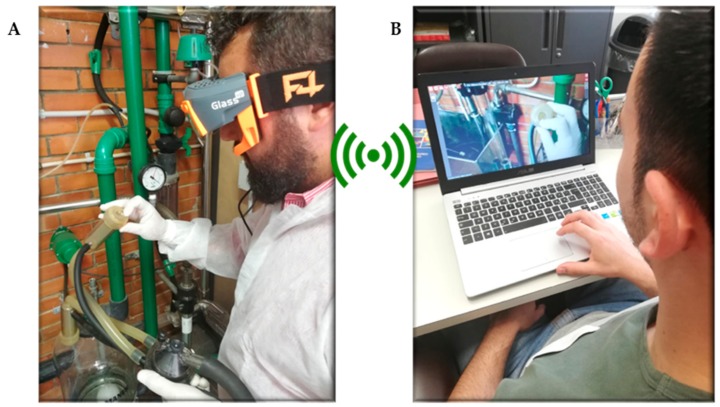
Remote assistance during milking machine inspection. (**A**) The emitting operator is inspecting the milking machine components, wearing augmented reality viewers, while working hands-free. (**B**) The receiving operator is assisting in real time to the emitting operator while working remotely.

The quality of the camera appears quite good during the video call or video recording. However, indoor and outdoor lighting may affect the readability on smart glasses, as observed during on farm investigations. The same considerations can be made for photo capture in different locations. Furthermore, the front light positioned above the field of view allowed improvement of video and photo quality in a low lighting environment (dark places). The lag-time during video-calls was steady over time, which allowed the comfortable use of this function for a long period and while moving to different working places. Specifically, using smart glasses for remote assistance may support unskilled operators during on farm activities, such as milking machine inspections (Figure 11).

The inconvenience of having an external joypad connected with a cable to the SG was balanced by the long battery life. In fact, the results demonstrate the F4SG works on an average of 7 h without any battery charge, both when scanning and video-calling. Most of the farmers work more than 8 h per day but SG for AR would barely be used, uninterrupted, all day. Therefore, the long battery life of this technology offers multiple applications in the agricultural and livestock field, ranging from remote assistance (video-call) to precision agriculture (QR code scan). Muensterer et al. [34] tested the battery life of a head wearable device in different conditions, obtaining between 8.5 and 12 h of activity on a typical clinical day, while in continuous video recording or videoconferencing the battery lasted about 30–40 min. Additionally, a systematic review conducted by Yoon et al. [43], on AR application for the surgeon, highlighted the disadvantage of the limited battery life of commercial head-up displays. In fact, among the seven wearable devices involved, three of those held a battery life up to 2 h and only one had a battery life ranging from 2 to 12 h.

Overall, using the available functions of F4SG (Table 5) enabled the operators to accomplish the typical on-farm activities involved in this study. In fact, the smart glasses for augmented reality have proven to be a valuable and integrative tool in accordance with precision livestock farming principles.

In this study, we explored the potential role that smart glasses for augmented reality (GlassUp F4) might have in agricultural and livestock farming. The F4SG are currently available on the market as AR goggles primarily designed for industrial applications. However, the outcomes of our study demonstrate that AR viewers may provide excellent opportunities in agriculture. The QR code scan function resulted in a helpful tool to support the breeders in flock management and also in feed procedures. The clear and fast readability of the information related to the single subject (e.g., animals and feed stocks), combined with the large number of readings that SG performed, allowed F4SG adoption even on large farms. In addition, the 7 h of battery life and good quality of audio-video features highlighted their valuable attitude in remote assistance, supporting farmers in the field. Many advantages can be offered to farmers from AR viewers, enabling real time file consulting, data collection, data sharing and remote assistance, all done while working hands-free. These are just some of the application of the smart glasses for AR in the precision livestock farming context. Nevertheless, some limitations have been found, in fact, there were no software applications during the study interval, specifically developed for the livestock sector. We are looking forward to evaluating competitor devices and providing feedback to support the forthcoming development of smart glasses designed for agricultural purposes.

## 5. Conclusions

The first evaluation aimed to discharge the battery during continuous scan-code function utilization, where the number of documents opened ranged from 860 to 1411. The second test was performed to evaluate the battery life during a video-call, assuming the use of smart-glasses for remote assistance or to have a visualization of the user’s point of view while working. The battery charge lasted, in both tests, for about 7 h. The battery life in each of five levels indicated by an LED on the joypad was variable and did not follow any definite trend both in the scan-code and the video call tests. The results showed that the only “level-0” in scan-code function lasted more than its equivalent video-call test (+55.4%) and the longer time was recorded in “level-3” for both cases. Knowing the duration time for each battery level, indicated by the LED, may be useful whether SGs are used occasionally, without necessarily having to be recharged. In fact, intensive use (more than 7 h) of SG may be rarely practiced in livestock farms.

## Figures and Tables

**Table 1:** Features of GlassUp F4 Smart Glasses.

Item	Technical Features
Processor	Cortex A9
Flash memory	8 Gigabytes
Operating System on board	Linux
Display	Color filter Active Matrix LCD (on right eye) Full color 640 × 480 pixel (VGA)
Sensors	Accelerometer (9 axis), gyroscope, compass, temperature and lux sensors
Connectivity	WiFi, Bluetooth
Camera	Full Color, 5 Mpixels, 15 FPS
Battery	Li-Polymer 5000 mAh
Operating temperature	5–35 °C
Weight (glasses)	251 g
International Protection (IP)	31

**Table 2:** Average scanning time (ST) and standard deviation (SD) with regard to QR code size. Minimum and maximum average scanning time for each QR size are reported.

QR Code Size (cm)	ST (s)	SD	Min ST (s)	Max ST (s)	Tot. scan (N°)
3.5	11.0 ^a^	5.7	4.1	34.7	1143
4.0	8.6 ^b^	3.8	4.2	33.9	1194
7.5	7.7 ^c^	2.8	3.9	28.3	1152

Values in the same column with diverse superscript letters are statistically different (*p* < 0.001).

**Table 3:** Average scanning time (ST) and standard deviation (SD) per operator. The minimum and maximum average scanning time for each operator are reported.

Operator	ST (s)	SD	Min ST (s)	Max ST (s)
a	9.4 ^a^	5.0	4.3	34.7
b	8.7 ^b^	4.1	3.9	33.2
c	9.1 ^a^	4.1	4.1	33.6

Values in the same column with diverse superscript letters are statistically different (*p* < 0.001).

**Table 4:** Battery life and smart glasses function used (mean of three repetitions).

	Battery Life (h)					
	Level 4	Level 3	Level 2	Level 1	Level 0	Total Battery Life
**Scan-code**	1.11 ± 0.41	1.89 ± 0.26	1.02 ± 0.45	0.98 ± 0.32	1.86 ± 1.12	6.87 ± 0.42
**Video call**	1.45 ± 0.33	2.13 ± 0.42	1.26 ± 0.29	1.33 ± 0.31	0.83 ± 0.58	7.01 ± 0.33

**Table 5:** Potential applications of smart glasses F4 in the livestock sector.

Smart Glasses F4 Functions	Applications	Examples
QR code scanning	Single subject identification	In livestock farms could help farmers to identify the animals and its productive data. Identify feedstock composition to improve feeding strategies. Retrieve fleet equipment information about history, maintenance, activity, etc.
VoIP call	Hands-free calling	The farmers could make hands-free calling while working, providing and/or receiving business and operative information on-the-go
Video streaming	Remote assistance while working	The farmer could share his point of view (live sharing) with a technician in real-time during maintenance procedures of equipment (e.g., milking parlor inspections)
Image acquisition	Photo capture and editing	During animal selection, farmers can take picture through the smart glasses to save the animal phenotypic relevant features. Photo acquisition may be also useful to underline the characteristics of spare parts of farm’s equipment. Photos may also be edited from the dashboard
Video-Audio recording	Video acquisition and saving	Recording and saving video off-line about different situations as system decision support tool; from animal diseases’ symptoms to systems’ anomalies.
Audio recording	Save notes and memorandum	The tractor driver could record voice annotation about on-farm procedures and draft by voice a checklist, while solving field operations.
File consulting	Audio, video, photo and text accessing during farm activities	Hands-free and immediate access to animal information (productions, health status, identification number, etc.). Tractor’s handbook consulting for maintenance support. This function allows to follow the on-screen instruction for problem solving or to recall and rapidly visualize the needed information.
